# Reference programme: Diagnosis and treatment of headache disorders and facial pain. Danish Headache Society, 2nd Edition, 2012

**DOI:** 10.1007/s10194-011-0402-9

**Published:** 2012-01-24

**Authors:** Lars Bendtsen, Steffen Birk, Helge Kasch, Karen Aegidius, Per Schmidt Sørensen, Lise Lykke Thomsen, Lars Poulsen, Mary-Jette Rasmussen, Christina Kruuse, Rigmor Jensen

**Affiliations:** 1 Department of Neurology, Danish Headache Centre, Glostrup Hospital, University of Copenhagen, Glostrup, 2600 Copenhagen, Denmark; 2 Department of Neurology, Department of Clinical Neurophysiology, Rigshospitalet, University of Copenhagen, 2100 Copenhagen, Denmark; 3 The Headache Clinic and Danish Pain Research Center, Aarhus University Hospital, 8000 Aarhus, Denmark; 4 Department of Neurology, University Hospital of Copenhagen, Bispebjerg Hospital, 2400 Copenhagen, Denmark; 5 Ny Tilemandsvej 4, Hammel, 8450 Favrskov, Denmark; 6 Department of Neuropediatrics, Juliane Marie Centret, Rigshospitalet, University of Copenhagen, 2100 Copenhagen, Denmark; 7 Research Unit of General Practice, University of Southern Denmark, J.B. Winsløws Vej 9A, 5000 Odense, Denmark; 8 Neurological Department, SVS, Finsensgade 35, Esbjerg, Denmark

## Abstract

Headache and facial pain are among the most common, disabling and costly disorders in Europe. Correct diagnosis and treatment is important for achieving a high quality of care. As a national organisation whose role is to educate and advocate for the needs of patients with primary headaches, the Danish Headache Society has set up a task force to develop a set of guidelines for the diagnosis, organisation and treatment of the most common types of headaches and for trigeminal neuralgia in Denmark. The guideline was published in Danish in 2010 and has been a great success. The Danish Headache Society decided to translate and publish our guideline in English to stimulate the discussion on optimal organisation and treatment of headache disorders and to encourage other national headache authorities to produce their own guidelines. The recommendations regarding the most common primary headaches and trigeminal neuralgia are largely in accordance with the European guidelines produced by the European Federation of Neurological Societies. The guideline provides a practical tool for use in daily clinical practice for primary care physicians, neurologists with a common interest in headache, as well as other health-care professionals treating headache patients. The guideline first describes how to examine and diagnose the headache patient and how headache treatment is organised in Denmark. This description is followed by individual sections on the characteristics, diagnosis, differential diagnosis and treatment of each of the major headache disorders and trigeminal neuralgia. The guideline includes many tables to facilitate a quick overview. Finally, the particular problems regarding headache in children and headache in relation to female hormones and pregnancy are described.

## Introduction

Headache conditions are among the ten most costly diseases in Europe and one in every three Danes have seen a physician at some point in their lives due to a headache. In a normal Danish general practice, more than 10% of patients have migraine and more than 5% suffer from a chronic, often incapacitating, headache. Due to the high occurrence, the overall socioeconomic costs derived from headaches are substantial and also cause 20% of the overall sickness absence recorded in the total Danish workforce. The quality of life of patients who suffer from headaches is also considerably reduced. Consumption of medication follows an increasing trend and the recent introduction of novel specific migraine medications and novel prophylactic pharmaceuticals contributes to a considerable need to establish a systematic and updated treatment strategy in Denmark. The overwhelming majority of Danes who suffer from headaches are currently being treated in primary health care and should continue to be so in future, but there is a growing need for clear guidelines on referral and organisation of specialist treatment of the more severe and rare headache conditions.

International general recommendations on the treatment of migraine and other primary headache disorders are now in place, and there is a substantial need to implement such guidelines in the Danish context.

In Denmark, patients with trigeminal neuralgia and other types of facial pain are typically treated by specialised neurologists, but even in this field there is a growing need for organisation and systematisation of treatment offers, particularly in the light of novel treatment strategies. This type of neuralgia is closely related to headache conditions and is therefore included in the present guidelines.

On this background, the Danish Headache Society established a workgroup with a view to revising the previous Danish reference programme on headache conditions from 1994. The present reference programme was prepared in relation to the general recommendations made by the previous Reference Programme Committee of the Danish National Board of Health and has been the subject of a hearing phase in the Danish Neurological Society and Danish Headache Society. In the present paper, the reference programme is translated into English and adapted to journal format. The original reference programme in colour can be downloaded for free at http://www.dhos.dk/index15.htm.

### Objective

The objective of this programme is to establish guidelines for the:

- diagnosis,
- organisation and
- treatment of the most frequently occurring primary headache disorders in Denmark such as migraine, tension-type headache, cluster headache and trigeminal neuralgia and to
- describe the “warning signs” of serious life-threatening and other secondary headache conditions.

### Introduction to the guidelines

The current guidelines include tables to increase usability and facilitate quick lookups in the context of a clinical working day. Consequently, the sections may be read separately and the primary points are repeated. The tables include ICD-10 and ICPC-2 diagnostic codes, e.g. Table 3 [G43.0/N89].

Following an introductory section with background and general information and a summary, focus is on the diagnostic process and on clinical investigation and organisation. In headache disorders and facial pain, the medical history is essential as the diagnosis is based on the history in the overwhelming majority of cases. A correct diagnosis is an essential prerequisite to correct treatment.

Next, the individual headache disorders are described in separate sections including characteristics, diagnosis, differential diagnoses, treatment and a list of references. Finally, we describe the specific problems related to children and adolescents and to women in relation to hormones, pregnancy and lactation.

A joint and comprehensive evidence assessment of the recommended treatments exists, but herein we have chosen not to state the evidence class to improve readability and reduce the amount of text. For further information, please refer to the recommended literature.

### Target audience

Any physician or other health-care professional who regularly sees patients with headaches and facial pain, primarily general practitioners (GP), junior physicians in training, neurologists and paediatricians working in practices and at hospitals and, finally, public sector decision-makers.

## Diagnosis and organisation

### Occurrence

Most of us have a headache occasionally and consider this a normal, temporary phenomenon. Headache is, however, a problem for approximately 40% of the European population. An average general practice (1,500 patients) comprises:

- 150 adults and 30 children with bothersome migraine;
- 65 adults and 10 children with daily headaches, a considerable share of whom have medication overuse headaches;
- 1 patient with cluster headache.

Table 1 summarises the primary types of headache and facial pain.

**Table 1:** 

Character	Possible diagnosis	Description	Sections
Acutely occurring headache	Subarachnoid haemorrhage among others	Hyperacute, severe headache ± neurological symptoms	7
Episodic headache	Tension-type headache	Pressing headache with no accompanying symptoms	4
	Migraine ± aura	Pulsating headache, physical activity associated with aggravation of the condition. Accompanied by nausea, photo- and phonophobia	3
	Cluster headache and others	Unilateral headache with ipsilateral autonomic facial symptoms	5
	Trigeminal neuralgia	Unilateral, stabbing pain lasting a few seconds	8
Chronic headache	Medication overuse headache	Use of headache medication for more than 10–15 days/month	6
	Chronic tension-type headache	Pressing headache with no accompanying symptoms or medication overuse	4
	Increased intracranial pressure including cerebral tumours	Frequently with increasing intensity, frequently with nausea and neurological symptoms	7

Any patient may have several types of headache and facial pain concurrently and a considerable number of headache conditions are secondary to other conditions. Some of these are serious and should be identified (see Sect. 7), but in general these conditions are relatively rare and comprise <1% of the patients seen with a headache in primary health care

### Taking a medical history on headache/facial pain

The medical history is crucial to the diagnostics in all primary headache conditions, facial neuralgias and medication overuse headaches (Table 2). No reliable, diagnostic tests exist. The medical history shall reveal any warning signs indicating the presence of a serious secondary headache.

**Table 2:** 

Useful questions	
How many different types of headache/facial pain do you experience? Take a separate history for each type!	
Developments over time	Why have you chosen to see a physician now?
	When did the pain start?
	How often do you experience the pain (episodically, daily and/or constantly)?
	How long does each attack last?
Character	Intensity of the pain experienced?
	Quality and type of pain?
	Where is the pain located and is it spreading?
	Accompanying symptoms?
Causes	Predisposing and/or trigger factors?
	Aggravating and alleviating factors?
	Familiar disposition for headache/facial pain?
Pattern of reaction	What do you do during an attack?
	How is your level of activity affected?
	Medication, which and how much?
General state of health between attacks	Full recovery or any symptoms between attacks?
	Preoccupation, anxiety or fear of new attacks and their causes?

Warning signals identified from the history or the physical examination which warrant further examination are:

- thunderclap headache (severe headache with sudden onset);
- headache with atypical aura (lasting more than 1 h or including motor symptoms);
- newly presenting headache in an HIV or cancer patient;
- headache/facial pain accompanied by fever or neurological symptoms;
- progressive headache lasting weeks;
- newly presenting headache in patients below the age of 10 years or above 40 years.

### Physical examination of a headache/facial pain patient

Physical and neurological examinations should be performed to exclude or confirm any secondary headache. The physical examination will generally produce normal findings in case of a primary headache. Attacks of cluster headache will produce physical findings including lacrimation, reddening of the eyes, ptosis and similar symptoms (see Sect. 5). In trigeminal neuralgia, pain trigger points can often be identified (see Sect. 8). Blood pressure and pulse should always be taken. CT/MRI scans are rarely indicated, but should be performed where the history or physical examination raise suspicion of a secondary condition.

### Diagnostic diary and calendar

**Fig. 1:**
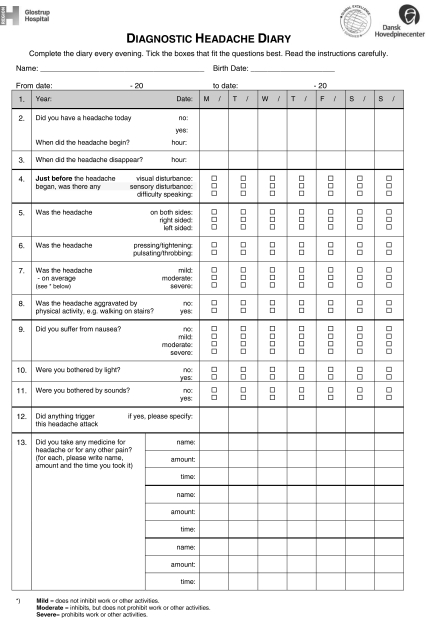
Headache diary. Available for download at http://www.dhos.dk

**Fig. 2:**
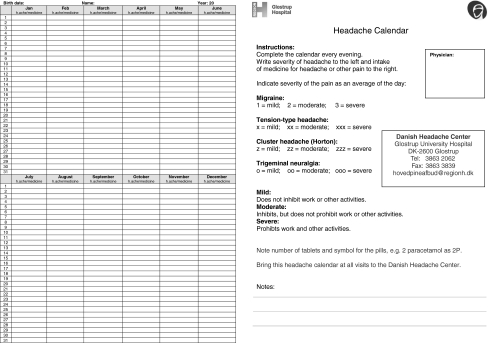
Headache calendar. Available for download at http://www.dhos.dk. Fold around the centre to A5 format; however, it may also be folded twice to A6 format to fit a pocket book or similar such book

Once a serious secondary headache has been excluded, the use of a headache diary (Fig. 1) for a minimum of 4 weeks and a headache calendar (Fig. 2) for a few months is highly recommendable.

Headache diaries and calendars may be downloaded and/or ordered at the Danish Headache Society’s Web page http://www.dhos.dk. These tools reveal to the physician and patient the headache pattern and also assist in identifying any trigger factors and medication overuse.

### Organisation of treatment in Denmark

**Fig. 3:**
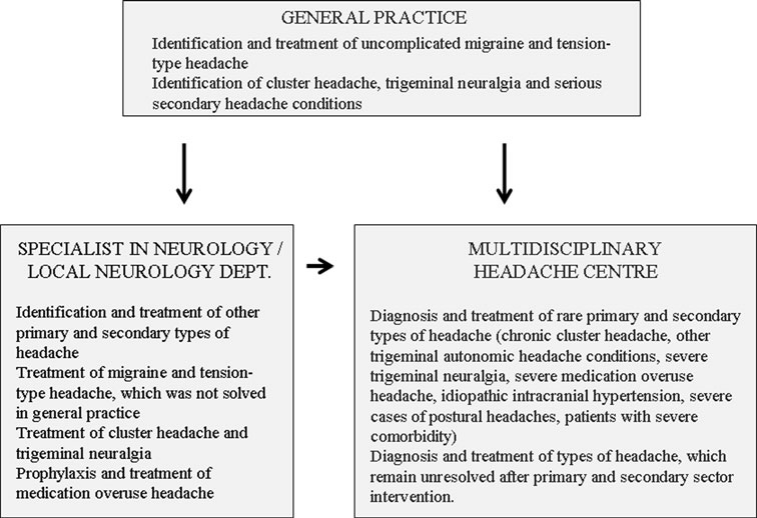
Organisation of the diagnosis and treatment of headache disorders and facial pain in Denmark

Diagnosis and treatment are generally performed at three levels (see Fig. 3). European guidelines have now been published on the organisation of headache conditions. In Denmark, diagnosis and treatment of headaches is and should generally be performed in primary health care, i.e. with the GP as the key health-care professional.

At the second level, diagnosis and treatment are performed by practising specialists of neurology or other related health-care staff with an interest/experience in pain conditions or by local neurology departments.

The tertiary level comprises headache centres, where medical specialists and cross-disciplinary staff specialised in headache conditions are responsible for diagnosis and treatment. This level is the highest level available in Denmark (see Fig. 3).

## Migraine with and without aura

### Diagnosis

The two most frequently occurring types of migraine are migraine with aura and migraine without aura. Many patients have both types. Migraine without aura presents as attacks lasting from 4 to 72 h and the typical characteristics are throbbing unilateral headaches of moderate to severe intensity with aggravation by routine physical activity. These headaches are typically accompanied by nausea, vomiting and phono- and photophobia (see Table 3).

**Table 3:** Classification of migraine without aura and typical aura with migraine headache [1]

1.1 [G43.0/N89] Migraine without aura
A. At least five attacks fulfilling criteria B–D
B. Headache attacks lasting 4–72 h (untreated or treated unsuccessfully)
C. Headache has at least two of the following characteristics:
1. Unilateral localisation
2. Pulsating quality
3. Moderate or severe pain intensity
4. Aggravation by or causing avoidance of routine physical activity such as climbing stairs
D. During headache at least one of the following:
1. Nausea and/or vomiting
2. Phono- and photophobia
E. Not attributed to another disorder
1.2.1 [G43.10/N89] Typical aura with migraine headache
A. At least two attacks fulfilling criteria B–D
B. Aura consisting of at least one of the following, but no motor weakness:
1. Fully reversible visual symptoms including positive features (e.g. flickering, spots or lines) and/or negative properties (i.e. loss of vision)
2. Fully reversible sensory symptoms including positive features (e.g. pins and needles) and/or negative features (e.g. numbness)
3. Fully reversible dysphasic speech disturbance
C. At least two of the following:
1. Homonymous visual symptoms and/or unilateral sensory symptoms
2. At least one aura symptom develops gradually over ≥5 min and/or different aura symptoms occur in succession over a period of ≥5 min
3. Each symptom lasts ≥5 and ≤60 min
D. Headache fulfilling criteria B–D 1.1 Migraine without aura begins during the aura or follows aura within 60 min
E. Not attributed to another disorder

Patients are symptom-free between attacks. The international headache classification [1] marked a substantial step forward for our understanding of the various types of headache, but also has the weakness of focusing on symptoms rather than patients. It may therefore be useful to focus on the overall differences between migraine and the most frequent differential diagnosis, tension-type headache, when the diagnosis is made. Furthermore, it is important to keep in mind that many patients suffer from migraine as well as tension-type headache. Table 4 summarises the typical characteristics of the two types of headache.

**Table 4:** Characteristics to distinguish between migraine and tension-type headache

	Migraine	Tension-type headache
Time pattern	Attacks lasting 4–72 h	Varies, from episodes lasting 30 min to a continuous headache
Headache characteristics	Frequently unilateral and pulsating. Aggravation by physical activity	Frequently bilateral and pressing. Normally no aggravation by physical activity
Intensity	Typically moderate to severe	Typically mild to moderate
Accompanying symptoms	Frequent nausea and/or vomiting, photophobia and phonophobia	None or only a mild nausea, photophobia or phonophobia

Approximately, a third of migraine patients have migraine with aura [2]. The aura phase consists of lateralised, reversible symptoms from the vision and tactile senses, such as flickering scotoma and sensory disturbances.

Transitory aphasia also occurs. Typically, symptoms develop gradually over minutes, every aura symptom has a duration of 5–60 min and several types of symptoms follow in a sequence (see Table 3). If aura includes motor weakness, the condition is classified as hemiplegic migraine. In migraine with aura, the headache phase frequently meets the criteria for migraine without aura and is then classified as typical aura with migraine headache (see Table 3). It should be noted that aura is not necessarily followed by headache, and that such headache does not necessarily meet the criteria for migraine without aura. In these cases, the migraine is diagnosed as typical aura with non-migraine headache or as typical aura with no headache [1].

### Background

Migraine conditions occur very frequently in Denmark with a lifetime prevalence of 16% [3, 4]. Migraine occurs in all age groups, also in children (see Sect. 10). More women than men have migraine. This is a particularly pronounced trend in migraine without aura, where the *M*/*F* ratio is 1:3 [3] (see Sect. 9).

Recent times have brought considerable improvements in our understanding of the mechanisms behind migraine. It is probable that migraine is a neurovascular disease in which a genetic disposition makes the brain of patients susceptible to a series of endogenous and exogenous trigger factors.

Migraine aura is probably caused by a transitory and slowly extending reduced neuronal metabolism, which typically starts occipitally, so-called cortical spreading depression [5, 6]. Migraine headache is probably caused by activation of nociceptors in the meninges and cerebral vessels and by secondary, increased pain sensitivity in the central nervous system [7].

### Clinical assessment and special assessment programme

Use of a headache diary is essential to reach the correct diagnosis, particularly to distinguish between mild migraine attacks and tension-type headache, and to exclude medication overuse headache.

Comorbidity, e.g. hypertension, asthma, severe obesity and depression, shall also be diagnosed and managed. Comorbid conditions are essential for the choice of prophylactic medication. Migraine is a benign condition, but women who have migraine with aura are at increased risk of stroke, even though their absolute risk is small (see Sect. 9). Typically, there is no need for paraclinical tests; see Sect. 2.

### Non-pharmacological treatment

- Generally, there is only limited evidence to support the effect of non-pharmacological treatment on migraine.
- In some patients, the following factors have a positive effect: information about the causes of migraine and about treatment options; a thorough examination allowing the patient to feel that he or she is safe and does not need to fear life-threatening disease; making the patient feel that he or she is being taken seriously.
- Identify and reduce, to the extent possible, any predisposing factors such as stress and depression/anxiety.
- Identify and eliminate, to the extent possible, any trigger factors, e.g. irregular lifestyle (e.g. poor sleep pattern or irregular food intake) and consumption of triggering foods such as red wine and some cheeses.
- Physiotherapy should primarily comprise instruction on how to maintain a correct work posture, correcting posture in general and instruction allowing the patient to perform active exercises at home. Physical activity may be beneficial.
- Biofeedback therapy has a documented effect on migraine [8].
- Behavioural therapy and cognitive therapy (stress and pain management) are probably effective, but offered only to a limited extent in Denmark.
- Controlled trials of the effect of acupuncture have yielded diverging results.

### Pharmacological treatment

Medical treatment is divided into acute treatment and prophylactic treatment.

#### Acute treatment

##### General guidelines

- No certain difference has been demonstrated between simple analgesics (paracetamol, NSAID and acetylsalicylic acid) alone or in combination with antiemetics and triptans [9]. Simple analgesics, i.e. in combination with antiemetics, are therefore first-line treatment [10]. Many of the patients who experience an insufficient effect of simple analgesics have good effect from triptans [10].
- Step-wise treatment is recommended in which each step comprises three treatments before progressing to the next step. Hereby, the most effective and inexpensive treatment is achieved [2, 10].
- The first step consists of simple analgesics and an antiemetic, if needed.
- The second step consists of triptans.
- Ergot alkaloids (ergotamine derivatives) are nearly obsolete due to the risk of serious side effects. These should only be used by specialists.
- Treatment should be initiated as early as possible during the attack [11]; triptans, however, should not be initiated until after any aura phase.
- Pharmaceutical treatment often has a better effect when combined with rest and/or sleep. If the patient has difficulty relaxing, benzodiazepine may be given, e.g. 5 mg diazepam.
- Please also refer to the section on prevention of medication overuse headache (see Sect. 6).

##### Simple analgesics and antiemetics

- It has been demonstrated that the following have an effect on migraine attacks: paracetamol, acetylsalicylic acid and various NSAIDs [2, 10]. See Table 5 for recommended doses.

**Table 5:** Acute migraine treatment, first step: simple analgesics and antiemetics with demonstrated effect in migraine attack treatment using the recommended initial doses [2, 10]

Analgesics	Initial dose (mg)	Antiemetic	Initial dose (mg)
Acetylsalicylic acid	1,000	Metoclopramide	10–20
Ibuprofen	400–600	Domperidone	20
Naproxen	500–750		
Diclofenac	50–100		
Tolfenamic acid	200		
Paracetamol	1,000		

These medications can typically be taken two to three times a day
- In case of accompanying nausea, simple analgesics may be given in conjunction with antiemetics to manage the vegetative symptoms and to increase resorption of the analgesics [12]. The following may be used: metoclopramide 20 mg suppository (or tablet 10–20 mg in case of aversion against suppositories) or tablet domperidone 10–20 mg (this last drug is frequently used in younger patients owing to its low risk of extrapyramidal side effects); see Table 5.
- Use of simple analgesics should not exceed 14 days/month to avoid medication overuse headache.

##### Triptans

- Triptans are generally alike with regard to effect and side effects [13], but the response of each patient to the various triptans may vary considerably [10]. Table 6 presents the triptans marketed in Denmark.

**Table 6:** Acute migraine treatment, second step: triptans available in Denmark (stated by date of marketing)

Triptan	Formulation	Comments
Sumatriptan	Tablets 50 and 100 mg	
	Nasal spray 10 and 20 mg	
	Suppositories 25 mg	
	Subcutaneous injection 6 mg	
Zolmitriptan	Tablets 2.5 and 5 mg	
Naratriptan	Tablets 2.5 mg	Less effective than sumatriptan
Rizatriptan	Tablets 10 mg	5 mg when used in conjunction with propranolol treatment
Almotriptan	Tablets 12.5 mg	Possibly less side effects than sumatriptan
Eletriptan	Tablets 40	80 mg allowed if 40 mg is ineffective
Frovatriptan	Tablets 2.5 mg	Possibly less effective, fewer side effects and a longer duration of effect than sumatriptan

An additional dose may be administered after a minimum of 2 h if the first dose has an effect and the headache returns. Generally, a maximum of two doses per day
- Patients who have no effect from one triptan may enjoy effect from another. To exclude any effect of triptans, the patient should, as a rule of thumb, have tried three different triptans, each during three attacks [2].
- Price differences between triptans are considerable.
- There is no evidence that the effect of orally disintegrating tablets or rapidly soluble tablets is any quicker than that of standard tablets. Nasal spray and subcutaneous injection act more rapidly than tablets.
- Triptans should be taken early in the attack (while the pain is mild) [14], but not during the aura phase, as they are ineffective in this phase [11]. To avoid overuse of triptans, it is essential that the patient is able to distinguish between migraine and tension-type headache.
- Some studies seem to indicate that a combination of a triptan and an NSAID is superior to each of the pharmaceuticals alone [15].
- Oral triptans may, in case of severe nausea/vomiting, be combined with the antiemetic metoclopramide [16] or domperidone; in some cases, non-oral administration is advantageous (nasal spray, suppository or subcutaneous injection).
- Approximately, 20–50% of patients experience migraine relapse within 48 h. An additional dose of triptan is normally effective in these cases. Migraine relapse may also be managed with NSAID.
- In case of lack of effect from triptans, repetition of the triptan treatment during the same attack is usually ineffective.
- Use of triptans should not exceed 9 days/month to avoid medication overuse headache.
- Common side effects include a sensation of pressure on the chest, nausea, distal paraesthesia and fatigue.
- Triptans are, among others, contraindicated in cases with uncontrolled hypertension, ischaemic heart conditions, previous cerebral infarctions and peripheral vascular diseases. Caution should be exercised when treating patients <18 and >65 years. However, sumatriptan nasal spray 10 mg is approved for use in adolescents aged 12–17 years. See http://www.medicin.dk for a comprehensive list.

#### Prophylactic treatment

##### General guidelines

- Prophylactic treatment is offered to reduce the frequency or severity of attacks.
- Prophylactic treatment should be considered [10] if
  - the number of attacks per month exceeds two;
  - attack medications have poor effect;
  - the patient’s quality of life is reduced considerably due to the migraine;
  - frequent or very long-lasting cases of aura occur.
- Thorough information to patients on the objective, any side effects and creation of realistic expectations about treatment effect is important.
- Prophylactic treatment is generally considered successful if the frequency or intensity of migraine is halved without introducing bothersome side effects [10].
- Medication overuse shall be managed before treatment is initiated (see Sect. 6)
- Prophylactic medication shall be chosen on the basis of the available scientific evidence for effect, adverse event profile and competing conditions. Tables 7 and 8 summarise the first-, second- and third-line drugs.

**Table 7:** Prophylactic migraine medication first-line medication [10]

Medicinal product	Daily dose
Beta-blockers	
Metoprolol	50–200 mg
Propranolol	40–240 mg
Anti-epileptic drugs	
Topiramate	25–100 (200) mg
Valproate	500–1,800 mg
Calcium canal blocker	
Flunarizine	5–10 mg

Recommended doses

**Table 8:** Prophylactic migraine medication, second- and third-line medications [10] and recommended doses

Medicinal product	Daily dose
Second-line	
Amitriptyline	10–100 mg
Naproxen	500 mg ×2
Bisoprolol	5–10 mg
Third-line	
Candesartan	16 mg
Lisinopril	20 mg
Acetylsalicylic acid	300 mg
Magnesium	360 mg
Riboflavine	400 mg
Methysergide	4–12 mg
Gabapentin	1,200–1,600 mg
Pitzotifen	1.5–3 mg
- Slow up-titration shall be used to minimise any side effects.
- A headache calendar shall be used to document any effect.
- Prophylactic treatment should be attempted for a minimum of 2–3 months at full dose, before it may finally be assessed if it has any effect (unless treatment is not tolerated due to side effects).
- In case of effect, discontinuation should be attempted every 6–12 months to confirm that the patient still needs the medication and that the medication remains effective.
- Lacking effect from one type of prophylaxis does not preclude the effect of other types of prophylaxis.
- There is no evidence to support an effect from a combination of several forms of prophylaxis.
- By ≥4 attacks per month or ≤10 days with headache per month, medication overuse should be excluded.

##### Beta-blockers

Beta-blockers with no autostimulating effect have a well-documented effect on migraine. Beta-blockers should normally be selected as the first among first-line drugs. Evidence is strongest for propranolol and metoprolol [17]. The typical dosage for metoprolol is 50 mg ×1 for a week and then 100 mg ×1, increased to 150–200 mg a day in case of lack of effect. The typical dosage of propranolol is 40 mg ×2, increased at a weekly interval to a maximum of 120 mg ×2.

Frequently, an effect is achieved at 120–160 mg daily. Once the dosage is established, a retard drug may be introduced to increase compliance. Furthermore, there is some evidence to support an effect of bisoprolol, timolol and atenolol [10]. Side effects include fatigue, dizziness, reduced physical capacity and cool extremities; see Table 7.

##### Anti-epileptic drugs

Topiramate [18] and valproate have a well-documented effect comparable to that of beta-blockers, but are generally associated with more side effects. The typical dosage for topiramate is 25 mg ×1, increased by 25 mg at 14-day intervals to 100 mg/day divided into two doses and, subsequently, dose adjustment if needed to 50–200 mg divided into two doses. Side effects include paraesthesias, sedation, dizziness, weight loss, kidney stones and a range of cognitive side effects. The typical dosage of valproate is 1,000 mg ×1 and in some cases a subsequent dose adjustment to 500–1,800 mg ×1 [10]. Side effects include dyspepsia, hand tremors, weight increase, affection of the liver, thrombocytopaenia and foetal malformations; see Table 7.

##### Calcium canal blocker

The effect of flunarizine is comparable to that of beta-blockers, but flunarizine is generally associated with more side effects [20]. The normal dosage is 10 mg ×1, but elderly patients should only receive half the dose [10]. Side effects include drowsiness, fatigue, weight increase, depression and unmasking of latent Parkinson disease; see Table 7.

##### Second- and third-line drugs

A range of pharmaceuticals are available, for which the evidence to support an effect is weaker or which have more side effects than the mentioned first-line medications. These pharmaceuticals are therefore categorised as second or third line; see Table 8. Several of these will normally only be used by specialists.

Naproxen dosage is 500 mg ×2 and this medication may also be used in short periods for treatment of menstrual migraine [21] (see the section on migraine and hormones). Candesartan has few side effects [22]. The typical dosage for candesartan is 8 mg ×1 for a week and then 16 mg ×1, if necessary increasing to 24–32 mg a day. Amitriptyline is particularly suitable if the patient also suffers from frequent tension-type headaches [10]. The typical dosage is 10 mg ×1 increased by 10 mg at 1-week intervals to 10–100 mg daily. The entire dose is given 1–2 h before bedtime. The typical dose needed to ensure an optimal balance between effect and side effects is 30–50 mg daily. Pizotifen should be given as 0.5-mg doses every third day to 1.5 mg nocte, possibly increasing to 1 mg ×3. Side effects include weight increase and fatigue.

## Tension-type headache

### Diagnosis

Tension-type headache is characterised by a bilateral, pressing pain of mild to moderate intensity. Such headache is not associated with the typical migraine characteristics such as aggravation by physical activity, vomiting or severe nausea and sensitivity to light and sound. There are three main types: (1) sporadic episodic tension-type headache, (2) frequent episodic tension-type headache and (3) chronic tension-type headache. The diagnostic criteria for tension-type headache are shown in Table 9.

**Table 9:** Classification of tension-type headache [1]

2.1 [G44.2/N95] Infrequent episodic tension-type headache
A. At least ten episodes occurring <1 day/month on average (<12 days/year) and fulfilling criteria B–D
B. Headache lasting from 30 min to 7 days
C. Headache has at least two of the following characteristics:
1. Bilateral localisation
2. Pressing/tightening (non-pulsating) quality
3. Mild or moderate intensity
4. Not aggravated by routine physical activity such as climbing stairs
D. Both of the following:
1. No nausea or vomiting (anorexia may occur)
2. No more than one of photophobia or phonophobia
E. Not attributed to another disorder
2.2 [G44.2/N95] Frequent episodic tension-type headache
*As 2.1 apart from:*
A. A minimum of ten episodes occurring ≥1 but <15 days/month on average during ≥3 months (≥12 and <180 days/year) and fulfilling criteria B–D
2.3 [G44.2/N95] Chronic tension-type headache
*As 2.1 apart from:*
A. Headache occurring on ≥15 days/month on average for >3 months (≥180 days/year) and fulfilling criteria B–D
B. Headache lasts hours or may be continuous
D. Both of the following:
1. No more than one of photophobia, phonophobia or mild nausea
2. Neither moderate/severe nausea nor vomiting

### Background

Episodic tension-type headache which occurs no more than a few times a month rarely causes concern and is often the body’s warning signal following inexpedient strain, e.g. due to stress or unphysiological work postures. Frequent episodic and chronic tension-type headache may, by contrast, be very bothersome and may reduce quality of life considerably [23].

The mechanisms behind tension-type headache are not fully known, but in the episodic form, referred pain from the pericranial musculoskeletal tissues and stress probably play an important role. In patients with frequent and chronic tension-type headache, it was demonstrated that the central nervous system was hypersensitive to pain. This may be due to insufficient inhibition of incoming painful stimuli or a consequence of frequent painful input from the pericranial musculoskeletal tissue (central sensitisation) [24].

### Clinical assessment and special assessment programme

Use of a headache diary is essential to reach the correct diagnosis, particularly to distinguish between tension-type headache and mild migraine attacks and to exclude medication overuse headache.

Physical examination is important, partly to demonstrate that complaints are taken seriously, and partly to ensure the exclusion of more serious conditions. Such reassurance may have an independent beneficial effect, e.g. in patients who have been worried that they may have a brain tumour. The examination should include palpation of the pericranial musculature to identify any soreness to assess the degree of musculoskeletal tensions, the chewing apparatus for bite dysfunction and sinuses for sinusitis. Comorbid conditions, particularly depression, should also be diagnosed and managed. Many patients would like to have a neck X-ray performed, but imaging of the neck is only rarely indicated and only on specific suspicion of cervical pathology. For further information on the need for paraclinical tests, please refer to Sect. 2.

### Non-pharmacological treatment (see Table 10)

Treatment of tension-type headache is primarily based on non-pharmacological measures. These are based on sparse or no scientific evidence, and therefore the following recommendations are based on “expert opinion” [25].

**Table 10:** Non-pharmacological treatment of tension-type headache

Physical examination and reassurance
Exclude other underlying disease, e.g. depression or oromandibular dysfunction
Exclude overuse of analgesics
Inform the patient about disease mechanisms
Minimise, to the extent possible, trigger factors, e.g. stress and unphysiological work postures
Physiotherapy (daily exercise programme and posture correction)
Biofeedback
Stress and pain management

- Identify and eliminate, to the extent possible, trigger factors, e.g. stress and unphysiological work postures. Physical activity may be beneficial.
- Provide information on the factors causing tension-type headache. It may be explained that each headache episode can be caused by muscle tension or stress, while chronic headache may also be caused by a disturbance in the centres of the brain which regulate pain.
- Physiotherapy should primarily comprise instruction on how to maintain a correct work posture, correcting posture in general and instruction, allowing the patient to perform active exercises aimed at reducing musculoskeletal tensions in the home. Controlled studies seem to indicate that such measures have an effect [26].
- Behavioural and cognitive therapy (stress and pain management) is typically performed by psychologists. Treatment includes instruction in stress relief, biofeedback (EMG and temperature) and cognitive techniques (restructuring of negative thoughts, among others). Treatment is focused on handling or coping with pain and stress. Such treatment is only offered in a limited number of places in Denmark. Controlled studies seem to indicate that such treatments have an effect [27]. Biofeedback alone has a documented effect on tension-type headache [8].
- Acupuncture is a frequently used measure. Controlled trials of the effect of acupuncture on tension-type headache have yielded diverging results [28].
- Manipulation of the cervical spine and blocking of the greater occipital nerve (n. occipitalis major) showed no effect in the few controlled studies performed.

### Pharmacological treatment (see Table 11)

#### Attack treatment

The effect of mild analgesics during the individual episodes of tension-type headache is well established [29], but the effect is often limited in patients with chronic tension-type headache. The following treatments are recommended [25]:

**Table 11:** Pharmacological treatment of tension-type headache

Treatment of the individual episode (paracetamol, ASA, NSAID)
Avoid overuse of analgesics
Avoid opioids
Prophylactic treatment is considered in chronic tension-type headache when non-pharmacological treatment has insufficient effect and medication overuse has been excluded
Amitriptyline, mirtazapine and venlafaxine have a prophylactic effect
Be sure to inform the patient that antidepressants are given to increase the concentration of pain-inhibitory substances in the central nervous system and not to manage depression
Use a headache calendar to monitor treatment effect
Discontinuation of prophylactic medication should be attempted every 6–12 months to confirm that the patient still needs the medication and that it remains effective

- ibuprofen 200–800 mg,
- ketoprofen 25 mg,
- acetylsalicylic acid 500–1,000 mg,
- naproxen 250–500 mg,
- diclofenac 12.5–100 mg,
- paracetamol 1,000 mg.

Controlled studies seem to indicate that NSAIDs are more effective than acetylsalicylic acid, which in turn are more efficient than paracetamol [25]. Paracetamol is, however, generally the medication which is tolerated best, and many patients find that the effect of the mild analgesics is comparable. Consequently, the choice of analgesics should be made on the basis of effect and side effects in the patient in question. It is extremely important to assess if there is any effect at all, or if the analgesics are rather taken “automatically” (“to do something”), and to set boundaries to avoid overuse.

Use of simple analgesics should not exceed 14 days/month. Codeine and a range of combination medications should, to the extent possible, be avoided and should only be used 9 days/month to prevent medication overuse headache (Sect. 6). Opioids should be avoided.

#### Prophylactic treatment

Prophylactic treatment may be indicated in patients with chronic tension-type headache if the effect of non-pharmacological treatment is insufficient and when medication overuse has been excluded [25]. Several placebo-controlled studies have shown an effect of the tricyclic antidepressant amitriptyline [30, 31], which is first-line treatment in prophylactic treatment of chronic tension-type headache. The effect is independent of any concurrent depression.

The newer serotonergic and noradrenergic antidepressants mirtazapine (30 mg/day) [31] and venlafaxine (150 mg/day) [32] have both been reported to be effective in a single study. These medications may be used with advantage where amitriptyline has no effect or in cases with concurrent depression. The effect of mirtazapine was comparable to that of amitriptyline. No effect has been documented from treatment with selective serotonin reuptake inhibitors (SSRI), muscle relaxants or botulinum toxin [25].

General guidelines:

- Inform thoroughly about the mechanisms of action (particularly that antidepressants are not given on the indication of depression) and about side effects.
- Provide slow dose escalation to minimise any side effects.
- Use an adequately high dose.
- Monitor the effect achieved by using a headache calendar.
- Assess the effect after 1–3 months at the final dose.
- Attempt discontinuation every 6–12 months.

1. Amitriptyline tablets, 10–75 mg.

Effect: 30% reduction of the headache versus placebo.

- ECG should be checked before treatment initiation.
- 10 mg ×1, increased by 10 mg/week until an effect is achieved or until side effects occur [25].

Maintenance dose, which ensures the optimal balance between effect and side effects, is typically 30–50 mg.

- The entire dose should be given 1 h before bedtime to improve sleep and minimise tiredness the following day.
- Typical side effects include, among others, dry mouth, fatigue, dizziness and weight gain.

2. Mirtazapine tablets, 15–30 mg.

Effect: 30% reduction of the headache versus placebo.

- 15 mg ×1, increased to 30 mg ×1 after a week.
- Administered approximately 1 h before bedtime.
- Typical side effects include, among others, fatigue, weight gain and dizziness.

### Summary

Tension-type headache is the most common of the primary headache types and comprises a substantial health problem in its frequent episodic and chronic forms. In episodic tension-type headache, referred pain from the pericranial musculoskeletal tissue and stress probably play an important role, while an altered central pain modulation is involved in the chronic form. A correct diagnosis is important, particularly differentiation between episodic tension-type headache and migraine. Comorbid factors, e.g. depression, and secondary headache, e.g. medication overuse headache, should be excluded.

Treatment is primarily based on non-pharmacological measures including information, minimisation of trigger factors, physiotherapy including posture correction and instruction about active exercises, and stress and pain management. The individual episodes may be treated with mild analgesics.

In patients with chronic tension-type headache, analgesics rarely have an effect and, therefore, prophylactic treatment with amitriptyline or mirtazapine may be indicated.

## Cluster headache (Horton’s headache)

### Diagnosis

Cluster headache, previously termed Horton’s headache, occurs in a series of attacks, so-called clusters, typically lasting 4–12 weeks. In between clusters, patients have symptom-free periods of highly varying lengths (from weeks to several years). Cluster headaches are severe, unilateral headaches with burning and drilling pain around or above one eye or temporally. In the majority of cases, the same side is affected in all attacks (but the pain can change sides). The pain typically has a duration of 45–90 min and may occur several times a day [33]. In some patients, the attacks occur at roughly the same time of day every day, and in others clusters occur at the same time of every year. The pain is accompanied by ipsilateral autonomic symptoms such as rhinorrhoea, lacrimation, ptosis, miosis and oedema of the eyelid as signs of parasympathetic hyperactivity and sympathetic hypoactivity. Patients are typically restless and agitated during attacks. Attacks often occur at night, typically 1–2 h after the patient has fallen asleep. Migraine-like symptoms such as nausea, photophobia and phonophobia may also occur [33]. There are two types: episodic (80–90%) and chronic (Table 12).

**Table 12:** Classification of cluster headache [1]

3.1 [G44.0/N90] Cluster headache
A. At least five attacks fulfilling criteria B–D
B. Severe unilateral orbital, supraorbital and/or temporal pain lasting from 15 to 180 min if untreated
C. Headache is accompanied by at least one of the following:
1. Ipsilateral conjunctival injection and/or lacrimation
2. Ipsilateral nasal congestion and/or rhinorrhoea
3. Ipsilateral oedema of the eyelid
4. Ipsilateral forehead and facial perspiration
5. Ipsilateral miosis and/or ptosis
6. A sense of restlessness or agitation
D. Attacks have a frequency from once every other day to eight per day
E. Not attributed to another disorder
3.1. [G43.01/N89] Episodic cluster headache
A. Attacks fulfilling criteria A–E for 3.1 cluster headache
B. At least two cluster periods lasting 7–365 days and separated by attack-free periods of ≥1 month
3.1.2 [G43.02/N89] Chronic cluster headache
A. Attacks fulfilling criteria A–E for 3.1 cluster headache
B. Attacks recur over >1 year without or with remission periods lasting >1 month

### Background

Onset of cluster headache typically occurs at 20–40 years of age and the condition has a prevalence of 80–100 per 100,000 persons [34]. Permanent remission is seen, but after 15 years with the disease, 80% of patients still have attacks [35]. Men have cluster headache five to seven times more frequently than women. Cluster headache is one of the most incapacitating types of headache [36].

The mechanism behind cluster headache is largely unknown. It is possible that cluster headache is partially caused by disturbances in the hypothalamus including secondary activation of pain-sensitive nerves [33].

### Clinical assessment and special assessment programme

In typical episodic cluster headache where the neurological examination is normal, imaging is not mandatory, but in atypical cases, treatment-refractory cluster headache and in chronic cluster headache, a cerebral MRI scan should be performed. Imaging is performed to exclude any space-occupying processes or midline malformations [37] or pathology at the sinus cavernosus, the hypothalamus and the pituitary gland [33]. The differential diagnosis regarding the other attack-based trigeminal autonomic cephalalgias (TACs) is primarily made on the basis of attack duration (see Table 13). Furthermore, a constant, unilateral form of headache exists which has accompanying autonomic symptoms, so-called hemicrania continua, which responds to indomethacin. These types of headache are very rare and will not be described further herein. For further information, see the EFNS guidelines [37].

**Table 13:** Clinical characteristics of the trigeminal autonomic cephalalgias (TACs) [33, 37]

	Cluster headache	Paroxysmal hemicrania	SUNCT
Epidemiology			
Sex ratio, F:M	1:3–6	2–3:1	1:8–12
Prevalence (%)	0.9	0.02	Very rare
Age at onset (years)	28–30	20–40	20–50
Pain			
Type	Drilling, throbbing	Drilling	Shooting
Intensity	Very severe	Very severe	Intense/very severe
Localisation	Periorbital	Orbital, temporal	Orbital, temporal
Attack duration	15–120 min	2–45 min	5–250 s
Attack frequency	1–8 per day	1–40 per day	3–200 per day
Autonomic accompanying symptoms	Yes	Yes	Yes
Effect of indomethacin	No	Yes	No
Attack treatment	Sumatriptan injections/spray, oxygen	None	None
Prophylactic treatment	Verapamil, lithium, prednisolone	Indomethacin	Lamotrigine, topiramate, gabapentin

*SUNCT* short-lasting unilateral neuralgia, headache attacks with conjunctival injection and tearing

### Non-pharmacological treatment

Generally, there is no effect of non-pharmacological treatment on cluster headache [37].

### Pharmacological treatment (see Table 14)

General recommendations:

**Table 14:** Pharmacological attack treatment and prophylactic treatment of cluster headache

Medicinal product	Dosage
Attack treatment	
Pure oxygen, inhalation	From 7 to 12 l/min
Sumatriptan injection	6 mg
Sumatriptan nasally	20 mg
Prophylactic treatment	
Verapamil tablets	240–960 mg
Prednisolone	75 mg initially
Lithium	According to serum values

- Treatment is generally a specialist assignment.
- Patients should receive medication against the acute attacks and also prophylactic medical treatment.
- Prophylactic treatment is the most important form of management and should be initiated as rapidly as possible when a new cluster presents.
- For the majority of prophylactic medications, the dose should be increased as quickly as possible.
- In case of total absence of attacks over a 14-day period (note that patients may have brief attacks with autonomic symptoms showing that the cluster has not ended), or when the patient senses that a cluster has ended, tapering of prophylactic medications should be attempted.
- Alcohol consumption may aggravate cluster headache and should then be avoided in cluster periods. No other trigger factors are known. Many cluster headache patients are heavy smokers.

There is no evidence that smoking cessation alleviates cluster headache.

#### Attack treatment

##### Oxygen inhalation

Inhalation of pure (100%) oxygen via a non-rebreathable facial mask with a flow between 7–10 and 7–12 l/min is an effective attack treatment. Inhalation should be performed sitting in an upright position. Such treatment is safe and has no side effects or contraindications. Approximately, 60% of patients experience considerable pain relief within 30 min after inhalation [37]. Transportable oxygen equipment (oxygen cylinder + mask) is supplied by private companies on prescription from a department of neurology. Such equipment is normally supplied on the day it is ordered. As this service is relatively costly, the patient should only have the equipment for periods with cluster headache. Patients should contact the oxygen company with a view to retrieving the oxygen cylinder once their cluster period ends. There may be regional differences in the rules concerning when oxygen cylinders should be returned; for instance, patients in the capital region are only required to hand in cylinders if the cluster-free periods normally exceed a duration of 1 year.

##### Triptans

Injection of sumatriptan 6 mg subcutaneous leads to complete pain relief in approximately 75% of patients within 15 min [38] and is the first-line treatment for attacks if oxygen is ineffective or unavailable. Sumatriptan nasal spray 20 mg [39] is also effective, but the effect is achieved more slowly. Oral triptan formulations generally take too long to gain effect [40]. Patients with cluster headache are at risk of developing medication overuse headache from frequent use of triptans. Common side effects include a sensation of pressure on the chest, nausea, distal paraesthesia and fatigue. Triptans are contraindicated in cases with uncontrolled hypertension, ischaemic heart conditions, cerebrovascular conditions and peripheral vascular diseases. Also see Sect. 3.5.1.2.

##### Other medications

Standard analgesics are normally without effect.

#### Prophylactic treatment

##### Verapamil

Verapamil is the first-line prophylactic treatment of cluster headache [41, 42]. There is no scientific evidence on optimal dosing or on the use of standard tablets versus prolonged-release tablets. Treatment may, e.g. be initiated with tablets 80 mg ×3, increased by 80 mg/week.

The therapeutic dose is typically 240–480 mg a day. In case of lack of effect, the dose should be increased by 100 mg with a minimum interval of 1 week. The daily dose may need to be increased to 960 mg. ECG should be checked before treatment initiation and repeated for every 200 mg increase when reaching doses above 400 mg daily. Typical side effects are constipation, dizziness, ankle oedema, tiredness, exanthema, hypotension and bradycardia. Caution is advised in case of heart failure, AV block and when combined with beta-blockers.

##### Prednisolone

Prednisolone is used in episodic cluster headache to achieve a rapid effect until other prophylactic treatment has effect. Open studies have demonstrated an effect [43]. The effect is often excellent, but use is hampered by the severe side effects associated with long-term treatment. The typical dosage is 75 mg ×1 for 5 days followed by a 12.5 mg dose reduction daily until discontinuation.

##### Lithium carbonate

Lithium carbonate is primarily used in chronic cluster headache, but may also be used in the treatment of the episodic type. An effect has been demonstrated for both types of cluster headache in a series of open studies, while a placebo-controlled study failed to demonstrate any effect in episodic cluster headache [37]. The typical dosage is tablet lithium carbonate 300 mg (8.1 mmol) once daily increased after a week to one tablet twice daily. Subsequently, the dosage is increased by one tablet at 14-day intervals while monitoring serum lithium levels. A serum concentration from 0.4 to 0.8 mEq/l has been shown to be effective. Before treatment is initiated, ECG, weight and blood samples [potassium, sodium, creatinine, thyroid stimulating hormone (TSH)] should be checked. Serum lithium is checked weekly during the first few weeks and then every 2–4 months. Serum creatinine and TSH should be checked every 3–6 months. The typical side effects are nausea, loose stools, polyuria, hand tremors and weight increase.

##### Other prophylactic medicinal treatment

Methysergide and Ergocoffin may be used but are specialist treatments. There are some reports on the effect of topiramate, valproate and intranasal capsaicin.

#### Non-pharmacological prophylactic treatment

Blocking of the greater occipital nerve (n. occipitalis major) can interrupt accumulated attacks and may have a prophylactic effect for 4 weeks or more. An effect was demonstrated in a single controlled trial [44]. Diprospane solution for injection 2 ml + 0.5 ml lidocaine 20 mg/ml may be used. Diprospane 2 ml contains steroid corresponding to 10 mg of betamethasone with protracted effect and 4 mg of betamethasone with rapid onset. The block is placed halfway between the inion (protuberantia occipitalis externa) and the mastoid process, where the nerve is palpable. It is relatively uncomplicated to apply the block and not necessarily a specialist assignment. If an effect is achieved, a block will typically remain effective for 3–4 weeks. A minimum of 3-month interval should separate blocks to avoid connective tissue necrosis and alopecia. In special treatment-resistant cases, surgical insertion of electrodes which continually stimulate the greater occipital nerve bilaterally may be considered. This is only performed at a very few centres worldwide and currently not in Denmark. Furthermore, at a limited number of centres worldwide, deep-brain stimulation of the posterior hypothalamic region is offered [37].

### Summary

Cluster headache is a relatively rare type of headache, but it is very incapacitating and generally easy to diagnose. Attack treatments as well as prophylactic management generally have excellent effect. Consequently, it is essential that patients are diagnosed correctly so that they may receive sufficient treatment. Treatment is generally a specialist assignment.

## Medication overuse headache

### Diagnosis

Medication overuse headache (MOH) is a relatively frequently occurring secondary headache caused by overuse of analgesics and/or acute migraine medications [1]. MOH is primarily seen as a complication to a pre-existing headache condition.

MOH is defined as a chronic headache (>15 days/month) with concurrent intake of symptomatic medicine for 15 days/month or more for simple analgesics/NSAIDs, or intake of triptans, ergotamine or opioids for 10 days/month or more during a 3-month period (Table 15) [45].

**Table 15:** Diagnostic criteria for medication overuse headache [45]

A. Headache present on ≥15 days/month
B. Regular overuse for >3 months of one or more acute/symptomatic treatment drugs as defined under subforms of 8.2 medication overuse headache
1. Ergotamine, triptans, opioids or combination analgesics ≥10 days/month on a regular basis for >3 months
2. Simple analgesics or any combination of ergotamine, triptans or opioids for ≥15 days/month on a regular basis for >3 months without overuse of any single class alone
C. Headache has developed or markedly worsened during medication overuse

The typical MOH pattern is a gradually increasing headache developing over weeks to months, particularly with either an increased migraine frequency, awakening headache or characteristic shifts even within a single day from migraine to tension-type headache. The majority of prophylactic measures, pharmacological as well as non-pharmacological, are without effect where there is a concurrent overuse of acute attack medication [46].

When the medications in question are discontinued, the headache often recedes considerably in frequency and intensity and returns to the original episodic pattern. The headache then once again becomes susceptible to standard treatment, and MOH patients are generally easy to treat [47].

Medication overuse headache can also occur in patients who are predisposed to headaches when analgesics are taken for other indications, e.g. back pain or arthritis conditions [48].

### Background

MOH occurs in 1–2% of all adults and is increasingly seen in children and adolescents. MOH was originally described in 1951 by Peter and Horton of the Mayo Clinic, MN, USA on the basis of ergotamine overuse [49]. MOH has subsequently been described regularly in association with all analgesics including opioids and more specific migraine drugs such as triptans [47, 48, 50, 51]. Combination drugs containing codeine, caffeine and/or barbiturates generally carry a higher risk of MOH than simple analgesics and NSAIDs [47, 48, 50, 51].

The diagnostic distribution is very homogeneous across studies: 65–70% of the patients had migraine as their primary diagnosis, 27–30% tension-type headache and 8–20% had a combination of these or another type of headache.

The male/female ratio is 1:3.5, and the typical age at onset is 30–40 years [48, 53, 54]. The mechanism behind MOH is currently not known in detail, but upward adjustment of central serotonergic and dopaminergic regulatory transmitter systems is involved.

### Clinical assessment

The MOH diagnosis is made on the basis of a thorough interview and a general physical and neurological examination. It is often necessary that the patient keeps a headache diary including records of all medications taken for a minimum period of 1 month. The differential diagnoses to MOH are primarily chronic tension-type headache and migraine and combinations hereof. Naturally, other secondary headache causes should be excluded; see Sects. 1, 2 and 7.

According to the criteria, the patient must have experienced a minimum of 15 days with headache within the last 3-month period, but in practice these patients typically present after many years with daily headaches and frequent migraine-like attacks [48, 50, 53]. According to the criteria, the headache must have developed during the medication overuse period, or the primary headache shall have aggravated markedly, but it is often difficult to obtain this information as these chronic headaches may have persisted for a very long period of time.

### Acute treatment

#### Non-pharmacological

The essential elements of treatment are training and information along with support and treatment of any withdrawal symptoms (Table 16). The next step is discontinuation of all analgesics. Alternatively, attack medication may be reduced to a maximum of 2 days/week. It is unknown which approach works best. Patients should be informed that after discontinuation of analgesics, some develop withdrawal symptoms and rebound headache with migraine-like headaches and in some cases nausea, vomiting sleep disturbances, agitation, anxiety, nervousness, hypotension and tachycardia. These symptoms have a duration of 2–10 days depending on the type of medication causing the overuse: 2–3 days for triptans and 8–10 days for pharmaceuticals containing codeine or ergotamine [52, 53, 55]. As withdrawal symptoms recede, the patients may notice a spontaneous improvement of their headache over the following weeks to months. Many patients also experience a considerable general improvement of their condition as they are no longer affected by a daily medicine intake. During the process, support and understanding from relatives, the patient’s employer and GP are important during the entire withdrawal period. A notification of illness lasting 2–3 weeks will often be recommendable. It may be necessary to refer the patient to specialist treatment or to admit the patient to a neurology department in case of marked overuse, use of opioids, significant comorbidity or previous unsuccessful treatment courses [52, 53, 55]; see Table 16.

**Table 16:** Non-pharmacological treatment of medication overuse headache

The primary treatment elements:
Abrupt discontinuation of all analgesics and acute migraine medicine or a reduction of the intake of attack medication to a maximum of 2 days per week during a 2-month period
Training and information to patients as well as relatives and medical staff
Psychological support, information and treatment of any withdrawal symptoms such as severe headache, nausea, vomiting, sleep disturbances, increased sweating, agitation, anxiety, nervousness, hypotension and tachycardia for 2–10 days depending on the type and amount of medicine taken
Admission to a neurology department in cases with considerable medication overuse and/or significant comorbidity
A sick leave of 2–3 weeks is recommended
Follow-up with GP for 6–12 months
Spontaneous and marked reduction of headache over weeks to months. Many patients also experience a considerable general improvement of their general condition as they are no longer affected by a daily medicine intake

#### Pharmacological treatment

Support medicine may be needed during the first 1–2 weeks. The following may be used:

- levomepromazine 12.5–25 mg maximally three times a day for a week followed by rapid tapering off or
- promethazine 25 mg maximally three times a day for a week followed by rapid tapering-off;
- metoclopramide suppositories 20 mg in case of nausea, maximally three times a day for a week, then discontinuation (see Table 17).

**Table 17:** 

Pharmacological treatment of medication overuse headache
Support medicine may be needed during the first week and the following may then be used:
• Levomepromazine 12.5–25 mg as needed maximally three times per day or promethaxine 25 mg x as needed maximally three times per day for a week followed by rapid tapering off (1–2 weeks)
• Metoclopramide suppositories 20 mg in case of severe nausea and vomiting
• Phenobarbital 100–200 mg × 2–3 for the first 4–5 days in case of severe withdrawal symptoms after discontinuation of opioids/combination medicines. After opioid overuse, methadone 20 mg may be needed and should then be tapered off over a 4-day period
After 2 months
• Initiation of prophylactic medication in accordance with standard guidelines depending on the type of headache
• Thorough information to the patient on the correct use of acute and prophylactic medical treatment
• Previously used medication, which during the medication overuse period had no effect, may now have effect
• Close follow-up at GP or specialist to avoid relapse into medication overuse
• Limited re-initiation of attack medication

### Prophylactic treatment

#### Non-pharmacological

The objective of the treatment is to achieve abstinence from analgesics or a reduction in the intake of attack medication to a maximum of 2 days/week during an 8-week period. Patients should be informed that improvements may occur gradually and be permanent. Hereby, the chronic headache and behavioural pattern is interrupted and the headache resumes the typical attack pattern or recedes completely. Relaxation, bed rest and plenty of fluids are recommended and patients often experience that attacks recede considerably and may be managed without analgesics. After the 2 months have passed, a restrictive intake of analgesics or migraine medications is again acceptable. Ongoing registration of medication intake in a headache calendar and frequent visits to the GP are important to prevent recurrence of MOH.

#### Pharmacological

In case of a high attack frequency, it is necessary to initiate standard prophylactic treatment and it should be stressed that preventive medicine which proved ineffective during the medication overuse period may now again have become effective [47]. The choice of medication depends on the primary headache type, previous adverse event profile and any comorbidity.

### Summary

MOH is a rather frequently occurring secondary headache condition caused by overuse of analgesics and acute migraine medication. The treatment course may be protracted and difficult to complete, but the result is very positive and previously treatment-refractory patients may present with significantly reduced attack frequencies and intensities after medicine review. MOH is a condition which in principle can and should be prevented through general information to patients and a restrictive prescription behaviour.

## Secondary types of headache

### Diagnosis

Secondary headache is defined as one which presents with a close temporal association to another condition which may cause the headache.

According to the ICHD-II [1], secondary headache is divided into the following groups:

- headache following head and neck trauma;
- headache related to cranial or cervical vascular conditions;
- headache related to non-vascular conditions;
- headache related to intake of substances or discontinuation of such intake;
- headache related to infections;
- headache related to homoeostatic disturbances;
- headache or facial pain in relation to dysfunction of the cranium, neck, eyes, nose, sinuses, teeth, mouth or other facial or cranial structures.

### Background

In addition to the previously mentioned primary types of headache, headache may also occur as the first symptom of serious life-threatening disease. Even though these serious conditions comprise far <1% of all types of headache, a newly onset severe headache commands extra attention and thorough diagnosis [56–58]. Where there is a close temporal relation to a head trauma or systemic disease, the development of the headache should be followed closely during admission or regularly by the GP.

In the general population, approximately 2% have secondary headache [56–58] and among these the vast majority suffer from medication overuse headache induced or maintained by overuse of analgesics or specific migraine medication [56, 57]. The prognosis is generally good, as the headache tapers off after reduction of medicine intake [59]; see Sect. 6. The temporal development of headache is the key to diagnosis [60, 61]. A “crack” in the head combined with maximal pain intensity after seconds should always raise suspicion of a subarachnoid haemorrhage, while a progressive headache with subacute onset following a head trauma may be the first symptom of increased intracranial pressure and of an epidural haematoma which, if left untreated, may develop into a life-threatening condition over the course of few hours.

Subacute headache which has developed over 1–2 days and is accompanied by fever, malaise and possibly seizures may indicate a cerebral sinus vein thrombosis, meningo-encephalitis or a cerebral abscess. A headache with gradual onset which has developed over weeks to months accompanied by any epileptiform attack elements, personality changes, speech disturbances and/or hemiparesis may be caused by a space-occupying cerebral tumour or a chronic subdural haematoma.

### Special assessment programme

The medical history and physical neurological examination are fundamental elements in the identification of any serious secondary headache. A thorough neurological examination, including ophthalmoscopy and measurement of temperature and blood pressure, is essential for the diagnosis of serious secondary types of headache. In addition to the mandatory neurological examination, the assessment programme depends heavily on the onset and course of the headache.

In headaches with subacute or acute onset, the following may be indicated: CT or MRI scans of the cerebrum, MRI or CT angiography and venography [60, 61] and lumbar puncture with analysis of the CSF for cells, glucose and protein and pressure measurement.

In slowly progressing headache developing over weeks to months, there may be indication for CT or MRI scans of the cerebrum, and—if the neuroradiological examination is negative—for CFS analysis as stated above [60, 61].

### Clinical assessment

Warning signals identified from the history or the physical examination that warrant further examination:

- thunderclap headache (severe headache with sudden onset);
- headache with atypical aura (lasting more than 1 h or including motor symptoms);
- newly presenting headache in an HIV or cancer patient;
- headache/facial pain accompanied by fever or neurological symptoms;
- progressive headache lasting for weeks;
- newly presenting headache in patients below the age of 10 years or above 40 years.

It is important to acknowledge the symptoms of the severe secondary headache types. Please refer to general text books for further information, but we would like to stress the following forms:

Subarachnoid haemorrhage should be suspected in case of:

- thunderclap headache with acute onset;
- possibly initiated by a generalised seizure;
- possibly followed by affection of consciousness.

Temporal arteritis

- Typically occurs above 50 years of age;
- typical symptoms: headache and constitutional symptoms;
- tenderness on palpation of the temporal artery;
- jaw claudication (up to 40%);
- amaurosis fugax (approximately 10%), which may lead to blindness if left untreated;
- sedimentation rate exceeding 50 mm/h (only 3% have a normal SR);
- biopsy of the temporal artery tests positive in 50% of the cases;
- treatment should be initiated on clinical suspicion before any biopsy results are available.

Primary glaucoma

- Headache may be a symptom in narrow-angle glaucoma;
- rarely occurs before the age of 50 years;
- risk factors: family predisposition, female sex and myopia;
- the condition may present as ocular hypertension;
- painful red eye;
- semi-dilated pupil with no reaction to light;
- accompanied by nausea and vomiting;
- complaints of blurred vision and coloured rings around light objects.

Sinus vein thrombosis

- Gradually increasing headache with subacute onset is the presenting symptom in 70% of cases [64];
- fluctuating neurological symptoms:

1. paresis (60%),
2. papilloedema (30–60%),
3. meningitis (25–30%),
4. reduced level of consciousness (60%),
5. epileptic seizures (40–50%).

Idiopathic intracranial hypertension (other names: benign intracranial hypertension and pseudotumor cerebri) [63]:

- Rare cause of chronic headache in slim persons (1–2/100,000), but seen 20–40 times more frequently in overweight persons;
- may lead to permanent visual field defect or blindness if left untreated;
- typically seen in younger women;
- may be conditioned by severe obesity;
- the typical signs are headache, papilloedema, abducens paralysis (the affected eye cannot be moved temporally) and or pulsating tinnitus;
- on suspicion, admission is necessary (important differential diagnosis: sinus vein thrombosis) and neuroradiological assessment, i.e. measurement of the cerebrospinal pressure, which will have increased >25 cmH_2_O.

Other secondary types of headache

- Cerebral tumour;
- chronic post-traumatic headache;
- dissection of the carotid, vertebral or basilar artery;
- severe arterial hypertension and hypertensive encephalopathy;
- neuroinfection or severe systemic infection (including sinusitis);
- Arnold Chiari malformation;
- hydrocephalus;
- postlumbar puncture headache or spontaneous dura lesion.

### Treatment

Treatment depends on the diagnosis.

## Trigeminal neuralgia

### Diagnosis

In the majority of cases, trigeminal neuralgia (TN) is a unilateral condition with ultra-short stabbing pain located along one or more branches of the trigeminal nerve (Table 18). Numerous daily attacks may occur. The attacks are stereotypical in the individual patient. Trigeminal neuralgia most frequently presents in the second or third branch and is often misinterpreted as originating from teeth or sinuses. The pain is often triggered by stimuli such as chewing, washing of the face, speech, tooth-brushing, shaving and cold winds, but also occurs without any stimuli. Often, there are trigger points in the face. Pain may be intermittent. The condition may aggravate or recede completely for weeks to months and, in rare cases, years. Autonomic symptoms usually do not occur (see Sect. 5). The diagnostic criteria [1] for classic TN are shown in Table 19. Symptomatic TN is caused by a structural lesion (apart from neurovascular contact (a vessel, typically leading to the cerebellum, exercising pressure on the trigeminal nerve)). There is no safe way to distinguish between classic and symptomatic TN on the basis of pain characteristics or therapeutic effect. Atypical facial pain does not have the neuralgiform characteristics seen in TN, but is often a more constant, diffuse pain.

**Table 18:** Characteristics of trigeminal neuralgia

In the majority of cases, trigeminal neuralgia is a unilateral condition with ultra-short stabbing pain located along one or more branches of the trigeminal nerve
Onset is most often located to the second or third branch
Onset typically occurs after 50 years of age
The pain is often triggered by stimuli such as chewing, washing of the face, speech, tooth-brushing, shaving and cold winds, but also occurs without any stimuli. Often, there are trigger points in the face
Pain may be intermittent. Consequently, the condition may aggravate or recede completely for weeks to months, and in rare cases, years
Symptomatic trigeminal neuralgia may, e.g. be caused by space-occupying processes of the fossa posterior and by multiple sclerosis. If the cause is pressure from vessels of the cerebellopontine angle, the case is diagnosed as classical and non-symptomatic trigeminal neuralgia

**Table 19:** Diagnostic criteria of trigeminal neuralgia

13.1 [G50.00/N92] Classic trigeminal neuralgia
A. Paroxysmal attacks of pain lasting from a fraction of a second to 2 min, affecting one or more divisions of the trigeminal nerve and fulfilling criteria B and C
B. Pain has at least one of the following characteristics:
1. Intense, sharp, superficial or stabbing
2. Precipitated from trigger areas or by trigger factors
C. Attacks are stereotyped in the individual patient
D. There is no clinically evident neurological deficit
E. Not attributed to another disorder

### Background

Trigeminal neuralgia most frequently presents after the age of 50 years and affects slightly more women than men. Approximately, 200–250 new cases are seen in Denmark annually [64]. TN may be caused by compression of the trigeminal nerve, most frequently near its connection to the brainstem. Compression is frequently caused by a minor artery leading to the cerebellum. This causes demyelination of the nerve root and subsequently ephaptic impulse formation with cross-excitation of the neighbouring fibres (in plain English: short-circuiting of the nerve fibres). TN is furthermore seen in multiple sclerosis and in idiopathic cases [65].

### Clinical assessment

A thorough medical history and a detailed neurological examination are essential to reach the correct diagnosis. Sensory symptoms from the affected nerve branch and bilateral pain raise suspicion of secondary TN [66]. Age at onset of TN involving the first trigeminal branch or lack of effect of medicinal treatment cannot be used to identify symptomatic TN [64]. In patients with TN and no other symptoms, there is a symptomatic cause (in addition to neurovascular contact) in up to 15% of cases [64]. Therefore, a cerebral MRI scan should be performed to visualise any neurovascular contact and to exclude any secondary cause (e.g. multiple sclerosis, tumours).

### Non-pharmacological treatment

A limited number of patients report effect from acupuncture, but there is no scientific evidence to support an effect of such or other non-pharmacological treatment.

### Pharmacological treatment

#### Acute treatment

The effect of standard analgesics and opioids is usually very limited. In severe trigeminal neuralgia where acute intervention is needed, infusion of phenytoin or lidocaine may be attempted during admission.

#### Prophylactic treatment

Prophylactic treatment with anti-epileptics has a stabilising effect on the nerve and is effective in the majority of cases—at least initially [66] (Table 20). As TN often has a periodic course, the dose should be adjusted continually according to the effect achieved and side effects observed. A combination of the individual medications is common, but there is no scientific evidence to support the effect hereof [64]. There are no good scientific studies of pharmacological treatment of symptomatic TN [64], which is therefore treated in accordance with the guidelines of classic TN.

**Table 20:** Treatment of trigeminal neuralgia

Primarily prophylactic pharmacological treatment with anti-epileptics
Normally, weak analgesics and opioids have no effect
Spontaneous remission is frequent. If the patient has been pain-free for 3–4 weeks, gradual discontinuation of medical management may be considered
In case of acute aggravation, where the patient has problems ingesting food, attacks may be interrupted with a fosphenytoin or lidocaine infusion
In case of unsatisfactory effect from medical treatment, a decision should be made with regard to neurosurgical treatment (microvascular decompression or lesion treatment)
The decision on neurosurgical treatment should be made as quickly as possible to avoid development of a chronic neuropathic pain condition.

1. Carbamazepine.

*Effect* It is the only medication for which an effect has been demonstrated in several controlled studies. Approximately, 60–70% of patients achieve a minimum 50% pain reduction. The number needed to treat to achieve a substantial pain relief is 1.7–1.8 [64]. The effect is often hampered by side effects (the number needed to harm is 3.4) [64].

- The initial dose is 200–400 mg daily given as two doses, increased by 100 mg every second day until pain relief or significant side effects. The typical maintenance dose is 200–600 mg/day given as two or three doses.
- Daily doses of up to 2,400 mg may be necessary.
- Once the maintenance dose has been established, the patient should be treated with two daily doses given as prolonged-release tablets.

2. Oxcarbazepine may also be a first-line medication.

*Effect* Comparative studies seem to show that the effect is comparable to that of carbamazepine [64].

Oxcarbazepine is often tolerated better than carbamazepine (66).

- The initial dose is 300–600 mg daily given as two doses, increased by 300 mg every second day until pain relief or significant side effects.
- The typical maintenance dose is 600–1,800 mg/day given as two or three doses.
- Daily doses of up to 2,400 mg may be necessary.

3. Gabapentin may be used in combination with carbamazepine/oxcarbazepine or as monotherapy.

*Effect* No certain documented effect.

- The initial dose is 300 mg once daily increased by 300 mg daily until an effect is achieved or significant side effects are observed. The maximum dose is 3,600 mg daily divided into three doses
- The typical maintenance dose is 600–2,400 mg daily

4. Baclofen may be used to supplement carbamazepine/oxcarbazepine or as monotherapy.

*Effect* An effect was reported in two small studies.

- The initial dose is 10 mg daily given as two doses, increased by 5 mg every third day until pain relief or significant side effects.
- The typical maintenance dose is 30–80 mg/day given as three or four doses.

5. Lamotrigine may be used to supplement carbamazepine/oxcarbazepine or as monotherapy.

*Effect* A single study has demonstrated an effect in combination with carbamazepine/oxcarbazepine.

- The initial dose is 25 mg once daily for 2 weeks followed by 50 mg daily for 2 weeks, followed by 50 mg twice per day for 2 weeks. Thereafter, if needed, the dose may be increased by 50 mg/week until an effect is achieved or significant side effects are observed.
- The typical maintenance dose is 200–400 mg/day given as two doses, and in some cases 500 mg daily.

6. Valproate may be attempted as monotherapy or in combination with other medications.

*Effect* A few small studies seem to show an effect.

- The initial dose is 1,000 mg ×1 and, subsequently, if needed, dose adjustment of 500–1,500 mg once daily is made.

7. Phenytoin may be used to supplement carbamazepine/oxcarbazepine or as monotherapy.

*Effect* An effect was reported in uncontrolled studies.

- The initial dose is 5 mg/kg divided into two doses.
- The typical maintenance dose is 200–400 mg/day given as two doses.
- Inexpedient in long-term treatment due to serious long-term side effects.

8. Pregabalin may be attempted as monotherapy or in combination with other medications.

*Effect* An effect was reported in uncontrolled studies.

- The initial dose is 150 mg daily given as two doses, increased by 150 mg a week to a maximum of 600 mg daily.
- The typical maintenance dose is 150–600 mg/day given as two to three doses.

### Surgical management

In approximately 30% of patients, medical treatment is inadequate. In these patients, surgical treatment should be considered. It should be determined as quickly as possible if a patient can be treated adequately with pharmaceuticals or if surgery should be offered, as prolonged symptoms increase the risk of developing constant background pain and of loss of sensibility in the trigeminal area.

#### Microvascular decompression

Microvascular decompression is the most effective treatment [64]. In microvascular decompression, a retromastoid craniotomy is performed after which the vessel(s) compressing the nerve are dissected off. Approximately 90% of cases experience a good initial effect, 80% are pain-free after a year and 73% are pain-free after 5 years [64]. The perioperative mortality ranges from 0.2 to 0.5%, and up to 4% have serious side effects such as haematoma, CSF leakage or infarction. The most frequent side effect is ipsilateral hearing loss, which is seen in up to 10% of cases [64]. Dysaesthetic pain and loss of sensibility are seen in a small number of patients. There is no evidence to support the use of MRI scans to identify those patients who would benefit from microvascular decompression [64].

#### Lesion treatment

Lesion treatment may be used in patients who are unsuited for microvascular decompression surgery. Glycerol is injected under local anaesthesia to the trigeminal ganglion. Glycerol injection destroys the cell bodies in the ganglion. Thermocoagulation and balloon compression are other forms of lesion treatment offered in Denmark. Approximately, 68–85% of cases remain pain-free after a year, and 50% are pain-free after 5 years [64]. Lesion treatment may be repeated in case of lacking effect or relapse. Approximately, 4% develop severe dysaesthesia (anaesthesia dolorosa) [64]. Lesion treatment, then, is less invasive than microvascular decompression, but also has a lower success rate and a higher recurrence rate.

### Summary

In the majority of cases, trigeminal neuralgia is a unilateral condition with ultra-short stabbing pain located along one or more branches of the trigeminal nerve. The pain is often triggered by stimuli such as chewing, washing of the face, speech and tooth-brushing. Trigeminal neuralgia is primarily caused by compression of the nerve near its origin at the pons. The treatment consists primarily of prophylactic pharmacological treatment with anti-epileptics. In case of insufficient effect or unacceptable side effects, neurosurgical treatment using microvascular decompression or lesion treatment should be considered.

## Hormones and migraine

This section describes the particular conditions associated with migraine in relation to menstruation, hormonal treatment, pregnancy and breast-feeding.

### Diagnosis

Menstrual migraine is defined as migraine attacks occurring on the first menstrual day ± 2 days in a minimum of two out of three menstrual cycles [1]. Menstruation is defined as endometrial haemorrhage associated with either a normal menstrual cycle or discontinuation of added female sex hormone, e.g. from oestrogen-containing contraceptive pills and cyclic hormone therapy.

The overwhelming majority of women also have migraine attacks at points in time that are unrelated to their menstrual cycle. In such cases, migraine is classified as menstrually related migraine. If attacks occur exclusively in connection with menstrual periods, the condition is defined as pure menstrual migraine.

### Background

The occurrence of migraine is associated with the menstrual cycle. This is particularly pronounced for migraine without aura [67]. Migraine is equally frequent in pre-adolescent girls and boys, but approximately three times as many women as men have migraine after puberty. Migraine may be triggered by a sudden drop in the oestrogen level, but only if the level has previously remained high for several days [68]. This explains why migraine occurs more frequently at menstrual periods and less frequently during pregnancy. There is, however, no certain association with ovulation. Around menopause, some women experience an aggravation of their migraine [69]. After menopause, both the incidence [70] and the prevalence [71] of migraine decrease.

### Specific risk factors

The risk of cerebral infarction is approximately doubled in women with migraine with aura compared with the background population. If these women are under 45 years and are smokers or take oestrogen-containing contraceptive pills, the risk is considerably higher. As cerebral infarction occurs rarely in young persons, it is difficult to quantify the increase in relative risk [72]. However, it is now considered a fact that young women who have migraine with aura are at an increased risk of cerebral infarction. It is important to put this into perspective, as the absolute risk of cerebral infarction remains small in young women with migraine (18 cases per 100,000 female years).

In one study, an overweight of cardiovascular risk factors was found in patients with migraine [73], but the causes of the increased risk of cerebral infarction in women with migraine with aura remain unknown. Women above 45 years of age with migraine without aura do not have an increased risk of cerebral infarction [74]. Migraine with aura is thus a risk factor for cerebral infarction in women, and the risk is more pronounced in women below 45 years of age who are smokers and who take oestrogen-containing contraceptive pills [75]. In women suffering from migraine with aura it is essential, to the extent possible, to reduce other known and modifiable risk factors for cardiovascular disease (hypertension, hypercholesterolaemia, smoking, etc.) [76]. Contraceptive measures without systemic oestrogen contents should be used [1, 6, 7]. However, most experts do not consider that oestrogen-containing contraceptive pills are an absolute contraindication if there are substantial reasons to choose these [77]. For such treatment, contraceptive pills with the lowest possible oestrogen contents should be used, and the patient should be informed of the increased risk. Hormonal contraceptives only containing progesterone do not increase the risk of cerebral infarction [78]. Young women who have migraine with aura are strongly encouraged not to smoke. Women who have migraine with aura should be informed that they have a slightly increased risk of a blood clot in the brain, but that the risk is very limited if no other risk factors exist and if they abstain from smoking and from taking oestrogen-containing contraceptive pills. Consequently, for the overwhelming majority of these patients, migraine is a benign if bothersome condition with a good prognosis.

### The effect of hormone treatments on migraine

There are no well-executed prospective studies which show how contraceptive pills affect migraine occurrence. Two retrospective studies reported that 24–35% of women with migraine experienced an aggravation, 5% an improvement and 44–65% no change in their migraine during treatment with contraceptive pills [79]. In a recent, population-based study from Norway, an increased occurrence of migraine was found among women taking oestrogen-containing contraceptive pills [79]. The overall conclusion is that contraceptive pills often have no impact on the course of migraine, but when there is an impact, it is generally an aggravation.

Cyclic oestrogen treatment in connection with menstruation was reported to have some beneficial effect in a limited number of small studies, but such effect could not be confirmed in a recent placebo-controlled study [80]. When there is a need for hormonal substitution treatment, continuous treatment with transdermal oestrogen should be preferred, as in the majority of cases this does not aggravate migraine in contrast to oral hormone treatment [68]. Surgical procedures such as bilateral oophorectomy can generally not be recommended as these aggravate migraine in most cases [81].

### Treatment of menstrual migraine

The principles of non-pharmacological treatment and attack treatment in menstrual migraine do not differ from those of non-hormone associated migraine. Menstrual migraine, however, tends to be more difficult to treat than migraine which is not associated with menstruation [80].

Prophylactic treatment of menstrual migraine.

- Menstrual migraine may be treated by not stopping the use of contraceptive pills for several cycles, e.g. by taking contraceptive pills continuously for 9 weeks (instead of the standard 3 weeks), followed by a 7-day period without contraceptive pills [2]. If breakthrough bleeding occurs earlier, the pill-free period is taken when the bleeding occurs.
- Alternatively, cyclic prophylaxis may be used, i.e. 6 days of treatment starting 2 days before the first menstrual day (or before the pill-free phase for women who are taking contraceptive pills). Treatment consists of the following:
- NSAID, e.g. naproxen tablets 500 mg twice daily [21];
- OR frovatriptan tablets 2.5 mg twice daily. These should be given with caution due to the risk of medication overuse headache;
- OR brief dose increase of habitual prophylactic medication;
- OR magnesium tablets 360 mg once daily—it is not given as described above, but starting 15 days after the first menstrual day and ending when the next period starts;
- Cyclic prophylaxis should, as a minimum, be attempted for three cycles before concluding whether the treatment is effective.
- The scientific evidence to support these treatments is limited.

### Pregnancy

The patient is advised to be patient, as the migraine will end or recede considerably during pregnancy in 70% of patients. In the overwhelming majority of cases, migraine recurs after birth or at the end of the breast-feeding period.

Attack treatment

- To the extent possible, non-pharmacological treatment including relaxation, bed rest, ice packages, etc.
- If pharmacological treatment is needed, paracetamol is the first-line treatment.
- NSAID medications should be avoided.
- Sumatriptan may be used. There are data from more than 2,000 first-trimester exposures showing no signs of any excess incidence of unwanted foetal affection. A register study found three cases of oesophagus atresia in a group of 1,718 persons where the normal frequency was approximately 1/5,000. This may be a coincidence, but if there is an association, the risk is very low. The other triptans should be avoided.
- Caution is necessary in connection with the administration of metoclopramide in the final trimester. The antihistamine meclozine 25–50 mg ×2 may also be used against nausea, though not in the two final weeks before term
- Codeine medication must be avoided due to teratogenicity.
- Ergotamine is contraindicated due to its uterus-contracting effect.

Prophylactic treatment

- Note that migraine will often improve during pregnancy.
- Prophylactic treatment should be avoided if possible.
- Some experts state that propranolol, metoprolol, amitriptyline and magnesium may be used with caution [2, 10], but this remains controversial.

### Breast-feeding

Acute treatment

- To the extent possible, non-pharmacological treatment including relaxation, bed rest, ice packages, etc. may be used.
- If the above have been attempted and are insufficient,
- paracetamol and some NSAIDs may be used;
- sumatriptan may be used. Breastfeeding should be avoided for at least 24 h after intake of the other triptans. The milk for the next 24-h period may, e.g. be pumped out before taking any of the other triptans.
- Caution must be exercised with use of metoclopramide.
- ASA, benzodiazepines and ergotamine must be avoided.

Prophylactic treatment

- Pharmacological prophylaxis to the extent possible must be avoided.
- Valproate and amitriptyline may be used.

## Children and headache

### Diagnosis

Diagnosis of headache in children follows the ICHD-II criteria used in adults with some modifications (see footnotes of Table 21). This section focuses on the characteristics specific to children.

**Table 21:** Classification of headache in children

1.1 [G43.0/N89] Migraine without aura
A. A t least five attacks fulfilling criteria B–D
B. Headache attacks lasting from 4 to 72 h^a^
C. Headache has at least two of the following characteristics:
1. Unilateral localisation^b,c^
2. Pulsating quality
3. Moderate or severe pain intensity
4. Aggravation by or causing avoidance of routine physical activity such as climbing stairs
D. During headache at least one of the following:
1. Nausea and/or vomiting
2. Phono- and photophobia^d^
E. Not attributed to another disorder
1.2 [G43.1/N89] Migraine with aura
Children and adults share the same diagnostic criteria
1.3.1 [G43.82/N89] Cyclic vomiting
A. At least five attacks fulfilling criteria B–C
B. Episodic attacks, stereotypical in the individual patient, of intense nausea and vomiting
lasting from 1 h to 5 days
C. Vomiting during attacks occurs at least four times per hour for at least 1 h
D. Symptom-free between attacks
E. Not attributed to another disorder^e^
1.3.2 [G43.820/N89] Abdominal migraine
A. At least five attacks fulfilling criteria B–D
B. Attacks of abdominal pain lasting 1–72 h (untreated or unsuccessfully treated)
C. Abdominal pain with all of the following characteristics:
1. Midline localisation, periumbilical or poorly localised
2. Dull or “just sore” quality
3. Moderate or severe intensity
D. During the abdominal pain at least two of the following:
1. Anorexia
2. Nausea
3. Vomiting
4. Pallor
E. Not attributed to another disorder^f^
The attacks are not caused by another condition^f^
1.3.3 [G43.821/N89] Benign paroxysmal vertigo of childhood
A. At least five attacks fulfilling criterion B
B. Multiple episodes of severe vertigo^g^, occurring without warning and resolving spontaneously after a few minutes to hours
C. Normal neurological examination and audiometric and vestibular functions between attacks
D. Normal electroencephalogram
2. [G44.2/N95] Tension-type headache
Children and adults share the same diagnostic criteria

^a^In children <15 years of age, the duration of an attack may be 1–72 h; the duration of untreated headache in children with a duration below 2 h should, however, be documented in a headache diary

^b^Migraine headache is normally bilateral in small children; an adult pattern with unilateral pain is seen late in the adolescent period and in young adults

^c^Migraine headache is typically frontotemporal. Occipital headache in children, be it unilateral or bilateral, is rare and requires diagnostic caution, as the cause may be structural lesions

^d^In small children photo- and phonophobia can be deducted from how the children react

^e^Cyclic vomiting is an exclusion diagnosis. Medical history, physical and neurological examination should not raise suspicion of any other condition. Thorough diagnostic assessment is always necessary to exclude any other condition. Differential diagnoses: intermittent bowel obstruction (malrotation); kidney, liver or pancreas disease; increased intracranial pressure; poisoning; metabolic disease and epilepsy

^f^Medical history, and physical and neurological examinations should not provide signs of gastrointestinal or renal illness, otherwise such illness shall be excluded through relevant assessment programmes

^g^Often associated with nystagmus or vomiting; unilateral throbbing headache may occur in some attacks

### Background

Migraine in children differs from migraine in adults, particularly due to the following:

- shorter duration of attacks;
- more common bilateral localisation;
- distinct gastrointestinal symptoms.

The specific periodic syndromes which occur in children (cyclic tendency to vomit, abdominal migraine or benign paroxysmal vertigo) may be precursors to adult migraine, but these conditions are relatively rare. Other primary types of headache such as tension-type headache and cluster headache may also present in early childhood, even though onset is more common around adolescence, but the symptoms of these conditions do not otherwise differ from the symptoms seen in adults; see Sects. 4 and 5.

Headache caused by medication also occurs in children and adolescents, but is no different from that caused by medication in adults (see Sect. 5). Generally, children with headache should not receive attack medication more than two times a week.

### Clinical assessment

The headache diary is an important diagnostic tool. Generally, the following applies:

- Older children and adolescents can keep a headache diary with no problems.
- The majority of 7- to 11-year olds can themselves report pain frequency and intensity, but may need an adult to aide for the registration of other symptoms.
- In younger children, parents can observe and report their symptoms without influencing pain results [82], and the intensity of pain may be determined in children by use of a visual analogue scale [83].

All children with headache should have a complete physical and neurological examination performed. These examinations and any other diagnostic tests are primarily performed to exclude other causes of the child’s headache (for further information on differential diagnoses, please see Sects. 1, 2 and 7). The physical examination should also include measurement of blood pressure and pulse, and in some cases an eye examination.

### Treatment

Generally, children are treated according to the same treatment principles as adults. Migraine treatment studies only exist for children aged 12 years or more, and generally there is no scientific evidence to support treatment of children with other types of headache.

#### Non-pharmacological treatment

Non-pharmacological treatment should be attempted before pharmacological treatment is initiated, but scientific evidence is extremely limited. Generally, the following recommendations apply (Table 22):

**Table 22:** Non-pharmacological treatment of headache in children

Physical examination and reassurance
Exclude other underlying diseases, e.g. stress, psychogenic factors (problems at home, school or/and among friends), depression, anxiety, refraction anomalies, strabismus, eyestrain (computer work/games), oromandibular dysfunction, sinusitis, postural anomaly, passive/active smoking and inexpedient lifestyle
Exclude overuse of analgesics
Inform about disease mechanisms and make sure that the child and his parents understand these
Minimise or eliminate any trigger factors, e.g. stress or unphysiological work postures at school

- Identification and elimination/reduction of any relevant trigger factors.
- Interview about dietary habits and sleep patterns.
- Thorough information to children as well as parents.
- Posture correction, active exercises and correct work postures.
- Stress and pain handling, and in some cases biofeedback and stress relief instruction.
- Physical activity in the form of aerobics, swimming, jogging or cycling.

#### Pharmacological treatment

Pharmacological treatment of headache conditions in children is mainly based on studies in adults [84] (Table 23).

**Table 23:** Pharmacological treatment of headache in children

The acute and prophylactic medical treatment of migraine and tension-type headache are different
Treatment of acute attack of tension-type headache (paracetamol and/or NSAID)
Treatment of acute migraine attack (paracetamol, and/or NSAID combined with domperidone; alternatively sumatriptan nasal spray)
Avoid overuse of analgesics
Prophylactic treatment is considered in very frequent or severely incapacitating headache, where the effect of non-pharmacological treatment has proven insufficient and where acute attack treatment is insufficient
Generally, scientific evidence to underpin the effectiveness of prophylactic medical treatment in children with migraine and tension-type headache is very limited
Prophylactic pharmacological treatment should be given in adequate doses for a minimum of 3 months before any decisions can be made with regard to its effect
Treatment discontinuation should be attempted after 6–12 months to ensure that daily medication is still necessary

##### Acute migraine treatment

There is a documented effect of treatment with weak analgesics in the form of paracetamol or ibuprofen [85, 86] and specific treatment with sumatriptan nasal spray [87–89] or zolmitriptan tablets 2.5 mg [86]. Sumatriptan nasal spray is the only medication among the triptans which is approved for use in children (adolescents aged 12–17 years) in Denmark. Controlled trials seem to indicate that ibuprofen is superior to paracetamol [85]. Paracetamol is, however, generally the medication which is tolerated best, and many patients find that the effect of the mild analgesics is comparable. Consequently, the choice of analgesics should be made on the basis of effect and side effects in the patient in question.

Generally in children aged >2 years, the effect of the above analgesics are enhanced if supplemented by antiemetics. Among the antiemetics, domperidone is preferred owing to fewer extrapyramidal adverse reactions in the form of locomotive disturbances.

##### Acute treatment of tension-type headache

There is a documented effect of paracetamol.

### Prophylactic treatment

There is only indication for pharmacological prophylactic treatment of headache in children if non-pharmacological treatment is ineffective and/or the headache attacks are frequent (more than 3–4 attacks per month), prolonged and/or so severe that they affect the quality of life and/or function level.

#### Migraine prophylaxis

Beta-blockers and flunarizine have a documented prophylactic effect in children and adolescents [87–89], while the evidence for other forms of prophylaxis is sparse [87, 88]. If it is clearly necessary to attempt other prophylactic treatments, the following may be used in the stated order: amitriptyline, topiramate and valproate [87, 88]. Pizotifen and clonidine, in contrast, have no documented effect in children [88].

#### Prophylaxis of chronic tension-type headache

Prophylactic treatment with amitriptyline may possibly have an effect on chronic tension-type headache in children, but no placebo-controlled studies document this.
